# Baby Farming in England
*A lecture given to the Bristol Child Health Group on 12th March, 1959.


**Published:** 1959-10

**Authors:** Philip Evans

**Affiliations:** Children's Physician, Guy's Hospital, and Physician to The Hospital for Sick Children, Great Ormond Street, London.


### BABY FARMING IN ENGLAND*

PHILIP EVANS, M.D., M.SC., F.R.C.P.
*Children's Physician, Guy's Hospital, and Physician to The Hospital for Sick Children, Great Ormond Street, London.*

A many years ago,
When I was young and charming
As some of you may know
I practiced baby farming.

H.M.S. Pinafore.

This song from Gilbert and Sullivan's *Pinafore* was for me all I knew of baby farming until a few years ago I met a baby farmer. The encounter stimulated my curiosity, and made me search for information about the industry. It was difficult to find: there is no textbook of baby farming, no Baby Farmers' Annual to tell the tale of the vicissitudes of the trade. Most of the information I have found is in old newspapers and journals, but some can be gleaned from Parish Registers, and there is now an excellent chapter on it in Leslie Housden's book *The Prevention of Cruelty to Children* (1955)[25].

First of all, what is baby farming? I take it to be the acquisition and use of infants for profit. But there is ordinarily no profit in the baby himself, for Dean Swift's plan for Ireland[1] has never been adopted. He put forward in an 18th century political satire: "A modest proposal for preventing the children of poor people in Ireland from being a burden to their parents or country, and for making them beneficial to the public". Swift had "been assured . . . that a young healthy child, well nursed, is at a year old a most delicious, nourishing, and wholesome food, whether stewed, roasted, baked or ragout". He calculated that 120,000 children of poor parents were born every year, and that 20,000 should be reserved for breed, and that the remaining 100,000 should be offered for sale to persons of quality and fortune. "A child will make two dishes at an entertainment for friends; and when the family dines alone the fore or hindquarter will make a reasonable dish, and, seasoned with a little pepper and salt, will be very good boiled on the fourth day, especially in winter".

Swift pointed out that his scheme would provide money for the family, make men as kind to their wives in their pregnancies as they were to their mares in foal and cows in calf, increase the national revenue, greatly lessen the number of papists, and incur no danger of disobliging England. Eighteenth century humour is rather strong for twentieth century stomachs, so let us be serious.

In other types of farming, the aim is to buy stock, fatten and sell it, breed from it, shear it, milk it, or in some similar way to make enough to cover the cost and leave something over. But thin, bald, milkless infants are unprofitable creatures, so the procedure is to get paid for taking the baby, and then to spend as little on it as possible. The most economic way of doing this is to kill it. This agriculturally reasonable procedure has roused a lot of opposition and made people write to the papers. It must not allow us to forget the other baby farmers who made a poor but honest living out of it, those described in the *British Medical Journal*[2] in 1868 as the "large number of perfectly respectable persons who eke out an insufficient living by taking charge of 'nurse children . . .' they do their duty to the children, and . . . regard their nurslings in a number of cases with feelings of tenderness and affection. More than this, we have been brought face to face with numerous instances of adoption of children from pure motives of tenderness, and from that yearning for the young which is one of the most sacred instincts of humanity".

*A lecture given to the Bristol Child Health Group on 12th March, 1959.

We read in *The Wealthy Virginian* by Hoole Jackson[20] "Colonel Jack was placed in the care of a nurse soon after his birth, and it seems likely that his father was a man of means, as a good sum of money was deposited with the chosen foster-parent for young Jack's upkeep and education. The nurse had been well chosen and told only that the name of the child placed in her care was John. She treated him as well as her own son, whose name was also John, and, to distinguish them, she nicknamed her own boy Captain John, and because young Jack claimed that as the son of a gentlemen he deserved a superior rank, she named him Colonel Jack.

"Aye," she told him, "you *are* a gentleman, and shall be a colonel. Captains are nothing. Every tarpaulin, if he gets but to be lieutenant of a press-gang, is called Captain, but gentlemen are ever made colonels, and I have known colonels come to be lords and generals."

Beside Colonel Jack and Captain John there was another boy—whether a son of the nurse or a child taken in to rear is uncertain—but he was nicknamed Major John, and these three were brought up in a kindly manner by the nurse. Unfortunately for them, she died when Colonel Jack was only ten years of age. The Major was then eleven, and the Captain eight. They were turned loose on London's streets, but soon learned to lie comfortably in shop-doorways in summertime, and ash-holes in winter, as well as how to pick up a living. From a comfortable home they were flung into a world such as that of Oliver Twist, peopled with Fagins, Artful Dodgers, and kidnappers, in the environs of Rosemary Lane.[20]

We may wonder what the motives are which make people pay to get rid of their babies, for it might be, as an unfortunate baby farmer wrote on the night before she was hanged in Horsemonger-lane gaol, that parents who sought to get rid of their children were more to blame than herself, and that if there were not such parents there would be no baby farmers.[3]

Vastly the greatest reason is of course, the stigma and inconvenience of having an illegitimate child. The unmarried mother looses her respectability and most of her earning ability at once. This was the basis of the thriving maternity homes of the suburbs of London in the nineteenth century, where, if abortion could not be arranged, the baby could be removed to a baby farm the day it was born, and the mother could return unencumbered and outwardly untarnished shortly afterwards. "I'm a jokelar person, I am; and I says funny things and cheers 'em up. She needn't mind, and mustn't fret, and I'll see her all right. I'm the old original, I am, and have had hundreds."[2]

Often people who cannot afford to keep their children cannot pay for someone else to have them, although the Day Nursery survives, rather better run than in "those nurseries in the manufacturing districts which are absolutely necessary" (Dickinson declared in 1870)[4]. These were Day Nurseries in which the children were quieted with narcotics (usually laudanum), "the nurse using them in the day to lull noise, which would otherwise convert the nursery into a Babel, the mother giving the dose at night, so that she may be fitted for her work in the factory on the morrow". A hundred years ago, outside some of those dark Satanic mills, the mortality rate for children under five years old was five times as high as in some rural districts. Dr. Edward Lankester, a coroner, believed that so large a proportion of illegitimate children were killed before they were one year old that there were few left on whom to hold inquests after that age (Housden, 1955).

Yet another economic reason for baby farming should be mentioned, although safe milk and the rubber teat have abolished it. At one time wet-nurses were paid 25 guineas a year, or 10 guineas a quarter, and many young women had children in order to earn money in this way. If they were unwilling to abandon their babies as foundlings, which was a criminal offence, they could clear themselves legally and financially by paying a baby farmer a small sum to take them.[5]

*Early History*

Baby farming, I suspect, must have existed for centuries, but a deputation sent by the City of London to Edward VI (1547–53) proposing that fatherless children should be nursed in the country had little success. The practice seems to have increased suddenly in the reign of his sister, Elizabeth I (1558–1603). It was a feature of the English Renaissance, like the glorious birth of the English stage and its poetry, the music of Byrd, nationalism, navigation, the Navy and the exploration of the New World, tobacco, potatoes, and the Church of England as by law established. In the parish registers of Chesham, Bucks, there are no entries of the burial of nurse children between 1538 and 1574, but in the next 64 years there were 45, mostly from London. In only one case was the name of the father entered, in some even the mother's is absent. Most came from London. Similarly in Lewisham and Sydenham's registers, the entries start in 1576 and are frequent from 1603; in St. Olave's and Bermondsey 1590 and 1591 are the opening dates; in Mitchem, 1587 (possibly 1586)[6]. In the next century Alton, Hants, Stoke Poges, Bucks, and Orpington, Kent, came into the picture. Mentions are common in the registers of places near London, and occasional near other large towns like Bristol and Norwich.[7]

For comparison I have gone through Registers in two parishes in the Sussex Weald and found only one case, recorded in unusual detail because of its special nature, I suppose. Its date is 1759, twenty years after Captain Thomas Coram established the Foundling Hospital.

> "June 19. Mercy Crull, an Infant belonging to the corporation of the Governors and Guardians of the Hospital for the Maintenance of deserted Children nurs'd by Sarah Pierce in this Parish."[21]

The rural practice was for illegitimate children to stay with their mothers, burials being recorded in some such terms as these:

"1694. Mar. 31. Constance Bastard of that impudent whore Constance Mihels."[22]

A hundred years earlier Harris wrote:

"The Rector of a Parish twelve miles from London, with great relief of mind told me that his Parish, which was not small either in its Bounds or Number of Inhabitants and was situated in a very Wholesome Air, was, when he first came to it, filled with sucking infants, and yet in the space of one year he had buried them all, except two and one of his own whom being weak he had happily committed to my Care from his very Birth, and that the same Number of Infants being soon twice supplied, according to the usual Custom of hireling Nurses, from the very great and almost inexhaustible City, he had committed them all to their parent Earth in the very same Year . . ."[8]

Can one wonder that Blake[9] wrote in "Songs of Experience":

Is this a holy thing to see
 In a rich and fruitful land,
Babies reduced to misery
 Fed with cold and usurous hand?

*The 19th Century and After*

The first move against baby farming was started by that zealous reformer, Mr. Ernest Hart. In 1844, when a nineteen-year-old student at St. George's, he led the agitation which removed naval assistant surgeons from the cockpit to more fitting quarters.[10] In 1868, the forty-three-year-old editor of the *British Medical Journal*, ophthalmic and aural surgeon to St. Mary's Hospital, and Dean of its medical school, conducted an inquiry into baby farming in London and published the results in the *Journal*.[2] His method was simple: to answer advertisements in the newspapers for nursing homes where ladies "temporarily indisposed" could be received, and "baby can be left"; then either Hart or Dr. Alfred Wiltshire visited the homes, ostensibly on behalf of clients, and conversed with the proprietors. He also advertised, offering to pay for the adoption of a child, and visited some of the 333 people who answered.

His reports described a number of thriving little businesses, some doing a good trade in abortions, others (disliking the risks of such affairs) preferring to allow the ladies to go to term, or at least to seven months, so that the baby could be neglected and be supposed to have been stillborn or to have died at birth. Adoption and disposal of living babies were frequently mentioned, and Hart also visited some unsavoury farms. He found the fees paid to maternity homes to be about £50, which would have allowed a margin for the baby-farming fee of £5–£10. "As the matter now stands" (he wrote) "there is not the slightest difficulty in disposing of any number of children, so that they may give no further trouble, at £10 per head . . . Demand and supply seem very equally balanced; and at this time there is certainly a very brisk business."

Hart's disclosures attracted some attention. In March 1868 Mr. Vanderbyl asked the Home Secretary in Parliament if he intended to put the law in motion to suppress the practices described in the *British Medical Journal*. The Home Secretary thought, that if the published statements were true they reflected great disgrace on the nation, but observed that the offences described were crimes within the law, and that it was not his duty to act as public prosecutor. The attention of the police had been called to the subject since publication of the articles.[11] Four months later Lord Shaftesbury asked a similar question in the Lords, and got a like answer from the Duke of Marlborough.[3]

Apparently nothing more was done, or would be done until the police produced evidence of crime. In 1870 a musician named Cowen and a Police Sergeant Relf appear briefly, and in the first case sadly and courageously, as Victorian social reformers.[3]

Cowen's unmarried daughter was about to have a baby. Cowen answered an advertisement of Margaret Waters, a widow of 35, who kept a baby farm at Brixton. Each advertisement cost Mrs. Waters 9s., in each she offered to adopt a child for a £5 note. Cowen arranged for his daughter (whom he called Mrs. Willis) to give up the child three days after he was born. Cowen appears to have become suspicious of the establishment, and he probably told his doubts to Sergeant Relf. This officer answered an advertisement, gained admission to the house, found nine children drugged with opium and in a miserable condition, and also 92 pawnbroker's tickets, mostly for articles of infants' clothing. Five of the nine babies subsequently died, among them young John Walter Cowen. Mrs. Waters was put on trial for his murder, and her sister Sarah Ellis was charged with conspiring to procure money by false pretences. She pleaded guilty and was sentenced to 18 months' imprisonment.

Waters' trial opened on 21st September at Newgate. She admitted that she had been in the business for four years, having started it in order to earn enough money to pay a debt, and that she had had "say forty" children confided to her. The largest amount of milk ever in the house was three pints a day. After a while "the poor little sufferers appeared to lose the power of crying". She denied intent to kill, saying the babies had died of convulsions or diarrhoea. After three days the trial was over, she was convicted and sentenced to death.

During her confinement in the gaol of Horsemonger-lane (a new model prison built as a result of Howard's reforms; Leigh Hunt enjoyed its hospitality) she conducted herself with a propriety befitting her position, although sometimes she despaired and was unable to take food. On the evening before she died she wrote an account on "three sides of a sheet of foolscap" explaining her method of livelihood. This document, which I would dearly love to read, may still exist in some clergyman's attic. In the night she slept for two hours, but restlessly. She rose and dressed at 7 o'clock in the morning, and spent the rest of her time with the chaplain. At nine the bell began to toll, and presently she left her cell and was conducted to the prison yard.

She helped the executioner to pinion her, mounted the scaffold unaided, and allowed herself to be placed for the drop. As the executioner adjusted the white cap and noose, she uttered a fervent and touching prayer for forgiveness. While the words were still on her lips the bolt was drawn, and she soon ceased to live.

A contemporary medical comment[4] was: "The sentence of death passed upon Margaret Waters will not act as a deterrent . . . Baby farming and baby-murder will go on as heretofore . . . the baby-farmers will raise their prices because of greater risks." Nevertheless 1871 saw the setting-up of a Select Committee of the House of Commons, and in 1872 an "Infant Life Protection Act" was passed. This provided for the registration of baby farms, but did not prevent baby murder, for no registration was needed if a woman took only one child; she could act expeditiously, and if the child died take another with impunity.[12]

In London baby farming was supervised, as well as it could be under the Act, and in 1894–5 there were 38 registered houses, in which the infant mortality was 10 per cent, but another 510 children were known to be kept in unregistered houses, and their mortality was 20 per cent. London had by then become unpopular with baby farmers, owing to the vigilance of the Public Control Department of the London County Council.[12]

Outside London only two houses were registered, one in Manchester and one in Bath, and it was clear that the Infant Life Protection Act did not protect the lives of illegitimate children, except to a limited extent in these two towns.[12]

In 1895 Lord Onslow introduced a new bill in the House of Lords, but it was so altered in the Committee stage that its sponsors withdrew it. Following a change of government, the bill was re-introduced by Lord Denbigh in 1896. Its fate seemed uncertain. The *British Medical Journal* ran an excellent series of articles showing the ineffectiveness of the Act of 1872,[12] and then fortunately (one may say without being callous) Mrs. Dyer was arrested in Reading. She took about £10 for each child. The bodies of five were recovered from the Thames or its tributary the Kennet.[13] Mrs. Dyer's fate is recorded in the ballad "Mrs. Dyer, The Baby Farmer",[14] of which one verse will serve as an example:

Poor girls who fall from the straight path of virtue,
What could they do with a child in their arms?
The fault they committed they could not undo,
So the baby was sent to the cruel baby farms.

*Chorus:*

The old baby farmer, the wretched Mrs. Dyer,
At the Old Bailey her wages is paid,
In times long ago we'd ha' made a big fyer
And roasted so nicely that wicked old jade.

The new act passed into law in 1897. It did away with many of the loopholes of the first act, but some still existed. It still did not apply to homes taking only one child: in 1903 it was calculated that four-fiths of the children were still not protected and that in one northern union a similar proportion of the one-child homes were unsatisfactory.[15] Prosecutions under the Act seem to have been rare, although in 1903 a woman was sentenced to two years' hard labour for abandoning three children of unmarried mothers (who had paid her £15–£20 each) in railway stations and trains.[12]

It was possible to dispose of babies without coming up against the Act at all. Consider, for example, the case of Walters and Sachs.[17,18] In 1903 Sachs, a 29-year-old nurse, kept a maternity home in East Finchley, of which the newspaper advertisements said "baby can remain". Immediately after birth the infants were given to Walters,

54-years-old and also a nurse. Owing to the law's omission to supervise one-baby homes she had to act swiftly to make her living. She was under suspicion, probably between railway stations she felt a desire for coffee and a waitress saw her carrying a dead baby in a shawl. A police officer lodged in her house. One day she collected a baby, and gave it some chlorodyne. It died. She was followed by the policeman to South Kensington station (where, she stated, she was to meet the wife of the coast-guardman who wanted to adopt it,) and was arrested. Sachs and Walters were accused together at the Central Criminal Court. After a trial lasting two days the jury deliberated for forty minutes, found both women guilty of murder, and recommended them to mercy.

Mr. Justice Darling: On what grounds?

Foreman: Because, my Lord, they are women.[18]

They were sentenced to death. Walters wept but Sachs appeared unmoved.

According to the Act these women were not baby farmers: Sachs was the keeper of a maternity home and Walters an agent for transferring babies to foster mothers. They did not dispose of more than one baby at a time and so did not have to register the house—in any case they did not keep the infants alive for the statutory forty-eight hours; furthermore notification was only necessary if there was a down payment of less than £20, and Sachs and Walters charged £25–£30.[17]

In the new Children Act of 1908 this financial limit disappeared. Under the Act of 1948[19] the basis of the old Act was changed: it is the duty of the local authority not merely to regulate children's homes but to *assume the care* of children under certain circumstances, while it is equally the duty of the *parents to maintain contact* with the local authority and to contribute towards the child's support. Children's committees and children's officers must be appointed, but the clear placing of the responsibility on local authority and parents together, so that it is illegal to get rid of your child by passing a few five pound notes, is most important.

As you know, baby farming for profit has become rare. I wish we could say that it had disappeared, but as I told you I have met a baby farmer, and I have been told of a maternity home in Reigate from which, during the last war, babies were disposed of in the old way, but with some charge for maintenance (an incentive to keep the child alive) as well as a down payment, to homes in Yorkshire. Dickson[4] was not far wrong when he wrote in 1870: "Running from the highest to the lowest of the monied classes, a demand for nurses and nurseries will continue, and homes will still be open for the reception of infants, whose parents desire never to hear of them again . . . I believe it to be impossible for any law to wipe out this state of things altogether."

Nevertheless things are very much better. There are still many illegitimate births. The rate in England and Wales in 1871 was 17 per 1,000 unmarried women every year, but it had declined to 8 by 1911, and remained there (despite the war of 1914–18) until the 30's when it fell further to 5·5.[23] However, in the 1939–45 war it rose to nearly double and in 1955 was 9·4. These are the rates: as the population has increased by about two-thirds since 1871 there are nearly as many illegitimate babies born now as then. In England and Wales in 1955 there were 31,000 although a further 50,000 were probably conceived before marriage. 2,600 of the illegitimately born were legitimized by the subsequent marriage of their parents. Adoption has increased, in 1955 there were 13,000 legal adoptions, so that half the illegitimate children were legitimized or adopted. Children's Officers have to cope with the rest.

We here all like children for their own sake, but a falling birthrate makes governments value them more highly too, humanitarian feeling and political sense work together for the welfare of the infant. This might seem a threat to the continued existence of baby farmers but such resilient people need not repine. It is difficult to get a baby to adopt now, and in America the consumer shortage has reached such a pitch that large (but illegal) businesses have been built up to provide babies for a consideration.

When we open a newspaper nowadays we may wonder whether our active acquisitive civilization is progressing or just moving for moving's sake. In our care of children, even unwanted children, we can see some sign of forward progress, from darkness towards the light.

### REFERENCES

1. Swift, J. (1729), From *The Works of Jonathan Swift, D. D.*, ed. D. L. Purves, Edin. 1876.
2. *Brit. Med. J.* (1868). **1**, 75, 127, 174, 175, 197, 234, 262, 301.
3. Annual Register (1870). Part II, 116, 120.
4. Dickson, T. (1870). *Med. Pr. and Circ.*, **10**, 323.
5. Garrison, F. H. (1929). *History of Medicine*, Phila. and Lond., 419.
6. Forsyth, D. (1911). *Proc. Roy. Soc. Med.*, **4**, Pt. I, 10.
7. Cox, C. J. (1910). *The Parish Registers of England*, London, 64.
8. Harris, (1689). De morbis acutis infant., quoted by Still, F. G. (1931). *The History of Paediatrics*, Oxford.
9. Blake, W. (1794). "Songs of Experience", "Holy Thursday".
10. *Dictionary of National Biography*, Suppl., Oxford, 1901.
11. *Brit. Med. J.* (1868). **1**, 276.
12. *Ibid.* (1896). **1**, 489, 543, 617, 670, 747, 796, 1109.
13. *Ibid.* (1896). **1**, 993.
14. *The Week-end Book*, London, 1955.
15. Brodie-Hill, W. L. (1903). *The Times*, Jan. 24th, p. 15.
16. *Brit. Med. J.* (1903). **1**, 221.
17. *Lancet* (1903). **1**, 250.
18. *The Times* (1903), Jan. 16th, p. 2; Jan. 17th, p. 5.
19. H.M.S.O. Children Act, 1948.
20. *Country Life*, May 27th, 1954. "The Wealthy Virginian" by Hoole Jackson.
21. Bolney Parish Register.
22. Cuckfield Parish Register.
23. Registrar-General's Statistical Review, 1937, Vol. II.
24. Registrar-General's Statistical Review, 1946–50.
25. Housden, L. G. (1955). *The Prevention of Cruelty to Children*, London.

